# Social Introversion Personality Trait as Predictor of Internalizing Symptoms in Female Adolescents with Gender Dysphoria

**DOI:** 10.3390/jcm12093236

**Published:** 2023-04-30

**Authors:** Flora Furente, Emilia Matera, Lucia Margari, Elisabetta Lavorato, Federica Annecchini, Francesca Scarascia Mugnozza, Giuseppe Colacicco, Alessandra Gabellone, Maria Giuseppina Petruzzelli

**Affiliations:** 1 DiBraiN—Department of Translational Biomedicine Neurosciences, University of Bari “Aldo Moro”, 70100 Bari, Italy; flora.furente@uniba.it (F.F.);; 2 DiMePRe-J—Department of Precision and Rigenerative Medicine—Jonic Area, University of Bari “Aldo Moro”, 70100 Bari, Italy; emilia.matera@uniba.it (E.M.);; 3 Psychiatry Unit, Azienda Ospedaliero-Universitaria Policlinico di Bari, 70100 Bari, Italy

**Keywords:** adolescents, gender dysphoria, transgender, emotion dysregulation, body investment, protection, internalizing symptoms, depression, self-harm, suicide

## Abstract

The personality trait of social introversion refers to the individual inclination toward the inner/outer world. Moreover, adolescents who experience Gender Dysphoria (GD) can be involved in relationship conflicts with family, peers, and friends and experience stigmatization and rejection from society. This leads higher distress in females which are more sensitive to this kind of feelings. This leads in them frequently developing a negative sense of well-being and low self-esteem which increases their risk of internalizing symptoms. So, the aims of this study were: (1) to investigate the presence of significant differences in Social Introversion (SI) dimensions between an assigned-female at birth (AFAB) GD group and a cisgender female group both diagnosed with a depressive disorder, (2) to verify whether the two clinical groups may be characterized by different profiles of internalizing symptoms, (3) to investigate if the SI dimensions could promote the internalizing symptomatology. Our results confirmed the presence of significantly higher score in GD sample for MMPI-SI scale and subscales and showed no significant difference in depressive profiles. Lastly, SI could promote internalizing symptomatology in AFAB underlying a link between SI and depression in this condition which should be further investigated.

## 1. Introduction

Personality traits include relatively persistent patterns of thoughts, feelings, and behaviors, that could be affected by biological bases such as changes in brain structure or endocrine levels, as well as by experiences or social changes over the course of a person’s lifetime; even if this interaction is not still well understood and scientifically explained enough [1]. Introversion/extroversion is a personality trait that describes a person’s attitudes toward the inner/outer world. Introverted subjects are more confident with their inner world of thoughts and emotions and are more selective in social relationships; while extroverted subjects are more talkative, sociable, and inclined to appreciate the interaction with others. Although no one is completely introverted or extraverted, very introverted individuals are shy, withdrawn and lack communication and self-concept skills [2]. Some evidence suggests a neurobiological link between personality traits and risk for depression, but the mechanisms by which personality traits relate to axis I disorders remain poorly understood [3,4]. Personality traits may influence the occurrence of recurrent episodes of depression, impact on the sign and symptoms of the illnes, or reduce the severity of the depressive disorder [5,6]. There are evidences that extroversion in childhood may be a protective factor against the development of mental disorders [7], and that an inverse relationship with Major Depressive Disorder (MDD) could exist [8,9]. Therefore, introversion may represent an underlying heritable trait of etiologic significanceand it may be especially promising in its relationship to the phenomenology and outcome of depression; however, it is currently unclear whether depression underlies introversion or whether introversion develops depression [10]. There is an age-related variability in the correlation between mood disorders and personality characteristics. Maladaptive personality features can arise in younger individuals after the onset of a mood disorder or a complex personality disorder, whereas the advent of a severe mood disorder in later life can amplify the personality symptomatology [11,12,13]. According to data published by the World Health Organization, mood disorders are among the main causes of mortality and morbidity in children and adolescents and, in particular, female teenagers between 13 and 17 years old show the highest frequency of depression [14]. Several factors influence pediatric and adolescent mood disorders putting some adolescents are at greater risk for mental health conditions due to their living conditions, stigma, discrimination or exclusion, or lack of access to quality support and services [15]. In line with this statement, we know that many young individuals with gender dysphoria (GD), which is described as a “marked incongruence between their experienced or expressed gender and the one they were assigned at birth”, have a high risk of comorbidity with anxiety, depression, and personality disorders [16]. Negative feelings and low self-esteem, which can increase the risk of depression and social isolation, are common among adolescents with GD who can be often involved in relationship conflicts with family, peers, and friends in various aspects of daily life and who can experiment exclusion or rejection from society [17,18,19]. In spite this, little is known regarding the specific relationship between social introversion, internalizing symptoms, and depression in adolescents with GD. Given that depression is more prevalent among females during adolescence, and in recent years an increasing numbers of assigned female at birth (AFAB) have been referred to Gender Identity Development Service for children and adolescents [20,21,22], in this study we analyzed female adolescent patients with depressive disorders comparing a group of GD AFAB, at the beginning of their gender affirmation journey, to a group of cisgender females. We hypothesized that AFAB adolescents with GD may have a more significant introversion trait than cisgender adolescents, which may have an impact on the internalizing symptoms associated with depressive disorders. So, the first objective of the present study aims to determine whether there are significant differences in introversion personality trait between the two clinical groups, by comparing the scores of Social Introversion Scale—Si and Subscales (Si1—Shyness/Self-Consciousness Si2—Social Avoidance Si3—Alienation—Self and Others) of the Minnesota Multiphasic Personality Inventory-Adolescent (MMPI-A). The second is to determine whether the two clinical groups are characterized by different profiles of internalizing symptoms (Withdrawal/Depression, Anxiety/Depression, Somatic Complaints) assessed by the Child Behavior Checklist—Youth Self Report (CBCL-YSR). The last objective is to determine whether the Social Introversion Scale and its Subscales could serve as eventual predictor of internalizing symptomatology (Withdrawal/Depression, Anxiety/Depression, Somatic Complaints) in each clinical group. 

## 2. Materials and Methods

### 2.1. Participants

Between October 2019, and December 2022, we enrolled for this study adolescent patients (12–18 years old), referred to the Child and Adolescent Neuropsychiatry Unit, Translational Biomedicine and Neurosciences (DiBraiN), University of Bari, Italy. Subjects who met the DSM-5 diagnostic criteria for Depressive Disorders (disruptive mood dysregulation disorder, major depressive disorder, persistent depressive disorder, premenstrual dysphoric disorder, and unspecified/other specified depressive disorder) were included in the study. According to the presence/absence of feelings of gender incongruences, subjects were subdivided into two clinical groups: the first group consisted of cisgender females (F-cis), without feelings of gender incongruences, and the second group was composed by assigned female at birth with gender dysphoria according to diagnostic criteria of DSM 5 (GD-AFAB), at their first request for gender affirmation to the Service for Gender Dysphoria of Psychiatry Unit of the same University. Male sex, intellectual disability (ID) and autism spectrum disorder (ASD) were considered exclusion criteria for enrollment. The parents of the patients provided written informed consent, and the subjects also consented to the study after being informed of its aims. The study was approved by the Ethics Committee of the University Hospital of Bari.

### 2.2. Psychometric Assessment

All subjects underwent a clinical global assessment that included an anamnestic collection focusing on the personal and family history of psychiatric disorders and the administration of clinical psychometric self-reports such as the Self-Administered Psychiatric Scales for Children and Adolescents (SAFA) [23,24,25], The Difficulties in Emotion Regulation Scale (DERS) [26,27,28,29,30], and Body Investment Scale (BIS) [31,32,33,34,35]. In accordance with the objectives of this study, data on social introversion personality trait and internalizing symptoms were analyzed based on scores obtained from the Minnesota Multiphasic Personality Inventory-Adolescent (MMPI-A) [36] and Youth Self Report (YSR) [37].

The MMPI-A is an extensively used and translated instrument, designed to assess psychopathological personality traits among adolescents aged 14 to 18-years old, however, it may be administered to 12- and 13-years old adolescents under certain conditions. T score, standardized for boys and girls is derived from raw scores; T scores between 60 to 65 are classified as subclinical, whereas T scores above 65 are classified clinical. The development and psychometric properties of this instrument are described in Butcher et al., 1992 [36], and its scales have adequate reliability and validity. This paper is focused on the Social Introversion (SI) Standard Scale and its Subscales (Si1-Shyness/Self-Consciousness, Si2-Social Avoidance, Si3-Alienation).

Frigerio et al. [37] adapted the Italian version of YSR to include 112 items describing the presence of potentially internalizing and externalizing problematic behaviors. In addition, the items are classified into eight behavioral scales (Anxiety/Depression, Withdrawal/Depression, Somatic Complaints, Social Disorders, Thought Disorders, Attention Disorders, Delinquent Behavior, Aggressive Behavior). Normative cut-off scores are used to categorize variables into three levels (non-clinical, borderline, and clinical). Internal consistency was calculated Cronbach’s alpha values, ranging from good to excellent (0.66–0.87) [38]. Anxiety/Depression, Withdrawal/Depression and Somatic Complaints are used as indicators of internalizing symptoms in this paper. 

### 2.3. Statistical Analyses

All the variables were recorded in structured forms specifically for this research. Analyses were conducted using IBM SPSS Statistics 28 [39]. The sociodemographic and clinical characteristics of both groups, through frequencies, means and standard deviations (SD) were calculated using descriptive analyses and compared using chi-square test. For quantitative data, assumptions of normality were verified using the Shapiro-Wilk test according to the sample size and comparisons between groups were conducted using the unpaired T-student test or Mann-Withney test for independent samples according to distribution. Lastly, Binary Logistic Regressions were used to estimate the contribution of each domain of Social Introversion, independently, as predictor on Depression, Anxious and Somatic symptomatology in GD and Cisgender samples, using MMPI SI Scale and Subscales as independent variable and YSR Withdrawn/Depressed, Anxious/Depressed and Somatic Complaints Scales as dependent variable using T = 65 as cut-off for pathological scores, according to the psychometrics and standardization of both MMPI-A and YSR [36,40]. The level of significance was set at *p* < 0.05.

## 3. Results

With a total of 69 adolescents with a diagnosis of depressive disorder, the recruited cohort was divided into a GD sample (n = 33; mean age 15.42 ± 1.542) and a Cisgender sample (n = 36; mean age 14.81 ± 1.582). The sociodemographic and clinical features are outlined in Table 1.

The comparisons between diagnosis, emotional problems, and internalizing symptoms are presented in Table 2, while logistic regressions for both groups are presented in Table 3.

In particular, Body Investment and acceptance showed a statistically significant difference in BIS-I (*p* < 0.01). The comparison of Social Introversion dimensions (MMPI-SI and its subscales MMPI-SI1, MMPI-SI2 and MMPI-SI3) revealed that GD sample had significantly higher scores on the MMPI-SI scale (*p* = 0.02), MMPI-SI1 (*p* = 0.01) and MMPI-SI2 (*p* = 0.01) subscales. Otherwise, internalizing symptoms and emotion dysregulation did not differ significantly between subgroups. Moreover, according to Table 3, the univariate binary logistic regressions using the three dimension of internalizing symptoms of YSR as dependent variables and the MMPI-SI, MMPI-SI1 and MMPI-SI2 as independent variables shows that for GD sample MMPI-SI, SI1 and SI2 appear to be predictors for Withdrawal/Depressed dimension (*p* = 0.01, 0.02 e 0.02); MMPI-SI and SI2 emerge as predictors of Anxious/Depressed dimension (*p* = 0.03 e 0.01) and MMPI scale and all of the subscales are predictive of Somatic Complaints (*p* = 0.02, 0.02, 0.03 and 0.03) dimension. Otherwise, all variables were rejected as predictors for the three dimensions (Withdrawal/Depressed, Anxious/Depressed and Somatic Complaints) in the Cisgender sample.

## 4. Discussion

The main finding of this study was that AFAB GD patients with a diagnosis of depression had significantly higher scores on the Social Introversion (SI) Standard Scale of MMPI-A (total and subscales) than depressed cisgender female adolescents. Moreover, we discovered that the internalizing symptoms profiles, assessed by the CBCL-YSR, did not differ between study groups. Both of these results suggest investigating the eventual relationship between gender incongruence-related distress and social introversion as a personality trait... According to the diagnostic criteria of DSM-5 for GD, the perception of incongruence between one’s experienced/expressed gender and assigned gender at birth is associated with clinically significant distress or impairment in social, occupational, or other important areas of functioning [41]; thus, the clinical definition of GD implies that the distress related to gender incongruence is not only associated with a strong and persistent desire to live as a member of the opposite sex, but also with clinically significant distress or social and functional impairments [42]. Despite the fact that, in general, introverts are more prone to withdraw from social situations, we are aware of the complex relationship between social engagement and introversion [2]. Our study’s findings indicate that, despite comparable depressive and internalizing symptoms, adolescents with GD have higher rates of introversion than cisgender adolescents. This raises the question of how gender dysphoria and social introversion could be related. About the last objective of the study, we found that SI could be identified as predictor for internalizing symptomatology in AFAB adolescents with GD, but not in cisgender adolescents. Considering that introverts are more likely to reduce their approach behavior in social situations due to unfavorable expectation, we can assume that stigma, prejudice, and discrimination create a hostile and stressful social environment that may promote mental health problems, as proposed by the Minority stress model [43,44]. On the other hand, many gene variants and by gene-environment interactions may influence personality traits [45]. For instance, an emerging field of study is examining the co-occurrence between autism/autistic traits and gender diversity or GD [46,47,48], which has led to the hypothesizing of connections between these disorders. Moreover, autism and transgender identity in AFAB appear to co-occur at an even higher rate [49], so it is plausible to assume that the introversion personality trait found in GD female-assigned at birth adolescents could be in a clinical and etiopathological continuum with autistic traits and autism. Recent researches are underlying the importance to consider autism in a dimensional spectrum that ranges from neurotypical phenotypes to atypical ones [50] and, interestingly, it has been also suggested that, according to the Extreme Male Brain (EMB) theory [48,51] the presentation of autism in many cisgender girls and women differs from that of cisgender boys and men for their clinical features [49,50]. In addition, females have been found to be more sensitive to the interpersonal context than males, which may make females more sensitive than males to the adverse mental health effects of poor social connections [52,53]. Given that social isolation is a risk factor for depressive symptoms in early adolescence [54], and that both of the groups in our study were diagnosed with a depressive disorder, we wanted to examinate the relationship between the introversion levels measured by MMPI and internalizing dimensional symptoms. The results showed that only in the AFAB adolescents with GD could the Si total score and subscales have a predictive role on Withdrawal/Depressed, Anxious/Depressed and Somatic Complaints; so, we can assume that female-assigned at birth GD patients with depression demonstrate a higher trait of social introversion that can be explained not only by exclusion and stigmatization but also by an intrinsic misfunctioning of the social skills and social relationship abilities. This introversion trait associated with social pressure could play a role in the development of both gender-related and negative environment-related internalizing symptoms.

### 4.1. Strenght and Limitations

According to our knowledge, this is the first study evaluating the implications of social introversion personality traits on depression and its symptoms in female adolescents with and without GD. In contrast, the greatest limitation of this study is the small sample size, which is further constrained by the fact that in logistic regressions only partecipants who scored above the threshold on the MMPI scales were considered. Unfortunately, as result of the rarity of the condition in adolescence, it was difficult to recruit the depressed AFAB with GD. Due to this, the statistical power of the study is limited and It was not possible to apply the correct sample size estimation. Moreover, a comparison with other groups such as depressed assigned male at birth (AMAB) with GD, depressed cisgender males and control groups without depressive disorders could have added valuable information to this research. But, in selecting our sample, we hypothesized that assigned female at birth adolescents with GD, particularly at the beginning of their gender affirmation journey, share more characteristics with female cisgenders expressing depression; cisgender males with depressive disorders diagnosis are uncommon in this age range. Therefore, this research should be regarded as preliminary in this field.

### 4.2. Future Implications

Increasing attention is being paid to adolescent mental health, particularly in disciplines related to GD as younger and younger patients seek out specialized services. This necessitates finding a way to optimize recruiting methods so that they are less dependent on in-person visits. In fact, due to their introversion, anxiety symptoms, and dread of exclusion and stigmatization, it is possible that these individuals do not prefer to go outside. So, the Online Photovoice (OPV) method could be a way to bypass the issue in part, because it provides participants with the opportunity to express their own experience with as little manipulation as possible [55,56].

## 5. Conclusions

Clinical and scientific evidence suggests that the awareness of the perceived incongruence with the assigned at birth sex is frequently accompanied, in adolescent females, by significant distress manifested by anxiety, depression, and somatic complaints. When compared to a cisgender depressed group, however, the profiles of internalizing symptomatology are identical. In contrast, AFABs with GD demonstrate introversion in a more significant manner. Understanding better how social introversion and internalizing symptoms of depression interact in this vulnerable patient population can lead to a more complete and targeted diagnosis, as well as more effective treatments for gender affirmation services. 

## Figures and Tables

**Table 1:** Sociodemographic and Clinical features of both GD and Cisgender samples.

Sociodemographic and Clinical Features	GD (n = 33)	Cisgender (n = 36)	*p*-Value
**Age** (median (IQR))	16 (3)	15 (3)	0.11 ^b^
Personal and family history of psychiatric disorders			
Family History of psychiatric disorders n (%)	16 (45.7%)	22 (62.9%)	0.23 ^a^
Anxiety n (%) Depression n (%) Bipolar Disorder n (%) Psychosis n (%)	9 (27.3%) 11 (33.3%) 2 (6.1%) 5 (15.2%)	11 (31.4%) 11 (31.4%) 7 (20%) 3 (8.6%)	0.71 ^a^ 0.87 ^a^ 0.09 ^a^ 0.40 ^a^
Bullying suffered n (%)	16 (48.5%)	11 (30.6%)	0.13 ^a^
NSSI and Suicidal behavior			
NSSI behavior n (%) Suicidal ideation n (%) Suicidal acting n (%)	20 (60.6%) 19 (57.6%) 8 (24.2%)	15 (41.7%) 19 (52.8%) 9 (25%)	0.17 ^a^ 0.69 ^a^ 0.94 ^a^

^a^ X^2^ used for the comparison; ^b^ Mann–Whitney used for the comparison.

**Table 2:** Comparison of DSM-5 diagnosis, DERS, BIS, MMPI-Si and YSR scale and subscales between GD sample and Cisgender. Bold font is indicative of *p* < 0.05.

	GD Sample		Cisgender		
DSM-5 Diagnosis	n (%)		n (%)		*p*-Value
MDD	10 (30.3%)		12 (33.3%)		0.78 ^a^
PDD	11 (33.3%)		7 (19.4%)		0.19 ^a^
DMDD	4 (12.1%)		5 (13.9%)		0.83 ^a^
OSD	8 (24.2%)		12 (33.3%)		0.40 ^a^
	Mean (SD)	Median (IQR)	Mean (SD)	Median (IQR)	*p*-value
**D-TOT**	108.27 (1.54)	15 (3)	100.91 (29.15)	109 (42)	0.290
**BIS-C**	15.78 (3.61)	16 (5)	17.00 (5.02)	17 (6)	0.28
**BIS-T**	16.84 (5.44)	18 (9)	18.84 (5.68)	19 (8)	0.16
**BIS-P**	20.22 (5.20)	21 (6)	21.16 (6.12)	22 (10)	0.57 ^b^
**BIS-I**	13.59 (3.60)	14 (5)	21.23 (7.73)	23 (11)	<0.01 *
**MMPI-SI**	62.46 (13.54)	62 (23)	54.54 (9.61)	55 (16)	0.02 *
**MMPI-SI 1**	58.21 (12.91)	62 (20)	49.15 (10.66)	48 (20)	0.01 *
**MMPI-SI 2**	67.29 (14.01)	76 (25)	55.81 (11.79)	51 (16)	0.01 *^b^
**MMPI-SI 3**	45.54 (10.75)	45 (20)	45.46 (8.38)	45 (11)	0.98
**YSR-Anx/Depr**	68.73 (14.14)	67 (20)	65.59 (12.61)	61 (13)	0.34 ^b^
**YSR-Withdr/Depr**	68.93 (12.80)	66 (26)	64.04 (10.09)	64 (14)	0.17 ^b^
**YSR-Som Comp**	61.10 (9.47)	63 (16)	60.89 (9.85)	57 (16)	0.98 ^b^

* *p*-values < 0.05; ^a^ X^2^ used for the comparison; ^b^ Mann–Whitney used for the comparison; MDD: Major Depressive Disorder. PDD: Persistent Depressive Disorder. DMDD: Disruptive Mood Dysregulation Disorder. OSD: Other Specified Depressive Disorder.

**Table 3:** Binary Logistic regression using MMPI-SI and the subscale SI1 and SI2 using YSR-Withdrawn/depression, YSR-Anxious/depression and YSR-Somatic Complaints as dependent Variable for Gender Dysphoria group and Cisgender group.

**GD Sample**								
**YSR-Whitdrawn/Depression (Cut-Off > 65: n = 16)**								
	B	S.E.	Wald	gl	Sign.	Exp(B)	C.I. Inf.	C.I. Sup
**MMPI-SI**	0.19	0.07	6.77	1	**0.01**	1.21	1.05	1.40
Constant	−12.34	4.73	6.82	1	**0.01**	0.00		
**MMPI-SI1**	0.17	0.05	5.43	1	**0.02**	1.12	1.02	1.24
Constant	−7.18	3.09	5.41	1	**0.02**	0.01		
**MMPI-SI2**	0.12	0.05	5.55	1	**0.02**	1.13	1.02	1.25
Constant	−8.58	3.72	5.30	1	**0.02**	0.00		
**YSR-Anxious/Depression (Cut-Off > 65: n = 18)**								
	B	S.E.	Wald	gl	Sign.	Exp(B)	C.I. Inf.	C.I. Sup
**MMPI-SI**	0.09	0.04	4.81	1	**0.03**	1.09	1.01	1.18
Constant	−4.98	2.46	4.10	1	**0.04**	0.01		
**MMPI-SI1**	0.05	0.04	1.78	1	0.18	1.05	0.98	1.12
Constant	−2.26	2.03	1.24	1	0.27	0.104		
**MMPI-SI2**	0.09	0.04	6.19	1	**0.01**	1.10	1.02	1.18
Constant	−5.69	2.50	5.19	1	**0.02**	0.01		
**YSR-Somatic Complaints (Cut-Off > 65: n = 11)**								
	B	S.E.	Wald	gl	Sign.	Exp(B)	C.I. Inf.	C.I. Sup
**MMPI-SI**	0.28	0.12	5.62	1	**0.02**	1.32	1.05	1.67
Constant	−18.47	7.72	5.72	1	**0.02**	0.00		
**MMPI-SI1**	0.15	0.06	5.73	1	**0.02**	1.16	1.03	1.31
Constant	−9.51	3.96	5.77	1	**0.02**	0.00		
**MMPI-SI2**	0.13	0.05	4.97	1	**0.03**	1.13	1.02	1.27
Constant	−9.08	4.11	4.88	1	**0.03**	0.00		
**Cisgender Sample**								
**YSR-Whitdrawn/Depression (Cut-Off > 65: n = 12)**								
	B	S.E.	Wald	gl	Sign.	Exp(B)	C.I. Inf.	C.I. Sup
**MMPI-SI**	0.06	0.06	1.21	1	0.27	1.06	0.95	1.18
Constant	−3.62	3.08	1.38	1	0.24	0.03		
**MMPI-SI1**	0.01	0.04	0.10	1	0.75	1.01	0.93	1.10
Constant	−0.95	2.16	0.20	1	0.66	0.39		
**MMPI-SI2**	0.00	0.04	0.01	1	0.96	1.00	1.93	1.08
Constant	−0.41	2.23	0.03	1	0.85	0.66		
**YSR-Anxious/Depression (Cut-Off > 65: n = 14)**								
	B	S.E.	Wald	gl	Sign.	Exp(B)	C.I. Inf.	C.I. Sup
**MMPI-SI**	0.07	0.06	1.31	1	0.25	1.07	0.95	1.20
Constant	−4.47	3.39	1.74	1	0.19	0.01		
**MMPI-SI1**	0.03	0.05	0.41	1	0.52	1.03	0.94	1.12
Constant	−2.13	2.32	0.85	1	0.36	0.12		
**MMPI-SI2**	0.05	0.04	1.44	1	0.23	1.05	0.97	1.15
Constant	−3.57	2.47	2.09	1	0.15	0.03		
**YSR-Somatic Complaints (Cut-Off > 65: n = 8)**								
	B	S.E.	Wald	gl	Sign.	Exp(B)	C.I. Inf.	C.I. Sup
**MMPI-SI**	0.11	0.07	2.83	1	0.09	1.11	0.98	1.27
Constant	−6.57	3.71	3.15	1	0.08	0.00		
**MMPI-SI1**	0.07	0.05	2.23	1	0.14	1.07	0.98	1.18
Constant	−4.14	2.53	2.68	1	0.10	0.02		
**MMPI-SI2**	0.07	0.05	2.35	1	0.13	1.07	0.98	1.17
Constant	−4.39	2.60	2.85	1	0.10	0.01		

## Data Availability

The data presented in this study are available on request from the corresponding author.
